# Supplementary material for: Dissecting the genetic overlap between three complex phenotypes with trivariate MiXeR
Source: medRxiv. 2024 Feb 27:2024.02.23.24303236. Preprint. [Version 1] doi: 10.1101/2024.02.23.24303236 (PMC10925360; doi:10.1101/2024.02.23.24303236)
Supplement: Supplement 1 [file media-1.pdf]

Table S1. Parameter estimates for 16 independent MiXeR runs for the "core" simulated scenario.
The first column show the index of the optimization run (1-16), with the run having the smallest deviation from the median overlap pattern (as described in the methods section) marked with asterisk. Other columns show univariate polygenicities ($\pi_1^u, \pi_2^u, \pi_3^u$), discoverabilities ($\sigma_1, \sigma_2, \sigma_3$) and residual variances ($\sigma_{01}, \sigma_{02}, \sigma_{03}$) for Trait 1, Trait 2 and Trait 3 respectively; pairwise (bivariate) genetic overlaps ($\pi_{12}^b, \pi_{13}^b, \pi_{23}^b$), correlations of effect sizes within each of the three pairwise overlaps ($\rho_{12}, \rho_{13}, \rho_{23}$) and correlations between residuals ($\rho_{012}, \rho_{013}, \rho_{023}$) for Trait 1 and Trait 2, Trait 1 and Trait 3 and Trait 2 and Trait 3 pairs respectively; the genetic overlap between all three phenotypes (π_{123}).

Index	π_1^u	σ_2	σ_{01}	π_2^u	σ_2	σ_{02}	π_3^u	σ_3	σ_{03}	π_{12}^b	ρ_{12}	ρ_{012}	π_{13}^b	ρ_{13}	ρ_{013}	π_{23}^b	ρ_{23}	ρ_{023}	π_{123}
1	2.23E-03	8.40E-05	1.039	1.81E-03	1.04E-04	1.029	2.07E-03	9.01E-05	1.026	1.03E-03	0.000	0.000	1.08E-03	0.009	-0.012	8.86E-04	0.053	0.003	8.86E-04
2	2.20E-03	8.63E-05	1.034	2.01E-03	1.00E-04	1.017	1.91E-03	9.81E-05	1.031	1.07E-03	0.000	0.000	1.03E-03	0.003	-0.008	8.28E-04	0.068	0.002	8.28E-04
3	2.30E-03	8.56E-05	1.025	1.85E-03	1.03E-04	1.028	1.92E-03	9.96E-05	1.028	1.14E-03	0.000	0.001	1.02E-03	0.005	-0.013	7.18E-04	0.157	-0.004	7.18E-04
4	2.13E-03	9.07E-05	1.031	1.98E-03	9.90E-05	1.022	2.09E-03	9.18E-05	1.024	9.64E-04	0.000	0.000	1.16E-03	0.003	-0.009	8.67E-04	0.055	0.002	8.67E-04
5	2.27E-03	8.50E-05	1.032	1.94E-03	1.02E-04	1.023	1.93E-03	9.63E-05	1.034	1.14E-03	-0.004	0.005	1.11E-03	0.003	-0.008	8.29E-04	0.106	0.000	8.29E-04
6	2.13E-03	8.84E-05	1.037	1.87E-03	1.05E-04	1.021	1.91E-03	9.72E-05	1.031	1.09E-03	0.000	0.000	1.00E-03	0.002	-0.005	8.04E-04	0.096	0.001	6.45E-04
7	2.10E-03	9.00E-05	1.035	1.86E-03	1.04E-04	1.024	1.99E-03	9.25E-05	1.030	1.01E-03	0.000	0.000	1.05E-03	0.002	-0.007	7.72E-04	0.049	0.004	7.72E-04
8	2.21E-03	8.80E-05	1.028	1.88E-03	1.02E-04	1.029	1.95E-03	9.53E-05	1.031	1.10E-03	0.001	0.004	1.01E-03	0.001	-0.001	5.85E-04	0.208	0.000	5.85E-04
9	2.19E-03	8.81E-05	1.028	1.85E-03	1.00E-04	1.032	1.98E-03	9.27E-05	1.033	1.22E-03	0.000	0.000	9.99E-04	0.002	-0.006	8.49E-04	0.059	0.002	8.49E-04
10	1.96E-03	1.00E-04	1.030	1.85E-03	1.05E-04	1.025	1.90E-03	9.79E-05	1.029	9.67E-04	0.000	0.000	9.25E-04	0.003	-0.008	7.45E-04	0.097	0.000	7.45E-04
11	2.21E-03	8.58E-05	1.034	1.88E-03	1.02E-04	1.028	2.00E-03	9.49E-05	1.025	1.18E-03	-0.004	0.005	1.11E-03	0.003	-0.010	8.22E-04	0.091	0.001	8.22E-04
12	2.02E-03	9.83E-05	1.029	1.90E-03	1.04E-04	1.019	2.07E-03	9.30E-05	1.024	1.04E-03	0.000	0.000	1.11E-03	0.004	-0.013	8.08E-04	0.089	0.001	8.08E-04
13	2.11E-03	9.28E-05	1.030	1.88E-03	1.02E-04	1.025	2.05E-03	9.57E-05	1.023	9.90E-04	0.000	0.000	1.06E-03	0.002	-0.008	8.82E-04	0.047	0.003	8.82E-04
14	2.24E-03	8.86E-05	1.024	1.88E-03	1.05E-04	1.025	1.85E-03	1.01E-04	1.030	1.15E-03	0.000	0.000	1.01E-03	0.001	-0.006	7.46E-04	0.100	0.001	5.64E-04
15*	2.14E-03	8.82E-05	1.036	1.85E-03	1.04E-04	1.029	1.95E-03	9.86E-05	1.027	1.01E-03	0.000	0.000	1.06E-03	0.005	-0.012	8.08E-04	0.084	0.002	8.08E-04
16	2.35E-03	8.55E-05	1.020	1.89E-03	1.00E-04	1.030	1.96E-03	9.39E-05	1.035	1.14E-03	0.000	0.001	1.14E-03	0.006	-0.008	8.24E-04	0.106	0.001	8.24E-04

Table S2. Parameter estimates for 16 independent MiXeR runs for the "ring" simulated scenario.
The first column show the index of the optimization run (1-16), with the run having the smallest deviation from the median overlap pattern (as described in the methods section) marked with asterisk. Other columns show univariate polygenicities ($\pi_1^u, \pi_2^u, \pi_3^u$), discoverabilities ($\sigma_1, \sigma_2, \sigma_3$) and residual variances ($\sigma_{01}, \sigma_{02}, \sigma_{03}$) for Trait 1, Trait 2 and Trait 3 respectively; pairwise (bivariate) genetic overlaps ($\pi_{12}^b, \pi_{13}^b, \pi_{23}^b$), correlations of effect sizes within each of the three pairwise overlaps ($\rho_{12}, \rho_{13}, \rho_{23}$) and correlations between residuals ($\rho_{012}, \rho_{013}, \rho_{023}$) for Trait 1 and Trait 2, Trait 1 and Trait 3 and Trait 2 and Trait 3 pairs respectively; the genetic overlap between all three phenotypes (π_{123}).

Index	π_1^u	σ_2	σ_{01}	π_2^u	σ_2	σ_{02}	π_3^u	σ_3	σ_{03}	π_{12}^b	ρ_{12}	ρ_{012}	π_{13}^b	ρ_{13}	ρ_{013}	π_{23}^b	ρ_{23}	ρ_{023}	π_{123}
1	2.25E-03	8.67E-05	1.026	2.02E-03	9.59E-05	1.023	1.92E-03	9.66E-05	1.033	1.25E-03	0.000	0.000	9.00E-04	0.002	-0.005	7.97E-04	-0.001	0.009	2.57E-05
2	2.47E-03	8.17E-05	1.017	2.01E-03	9.97E-05	1.016	2.03E-03	9.53E-05	1.025	1.23E-03	-0.001	0.005	1.03E-03	0.000	0.000	1.16E-03	0.000	0.000	3.74E-04
3	2.36E-03	8.46E-05	1.020	1.78E-03	1.12E-04	1.018	1.94E-03	9.96E-05	1.027	1.13E-03	-0.005	0.006	1.01E-03	0.001	-0.004	1.05E-03	-0.003	0.004	3.93E-04
4	2.26E-03	8.64E-05	1.024	1.94E-03	1.04E-04	1.015	2.00E-03	9.60E-05	1.026	1.21E-03	0.000	0.000	1.00E-03	0.001	-0.002	1.04E-03	-0.004	0.006	3.06E-04
5*	2.22E-03	8.57E-05	1.029	1.88E-03	1.09E-04	1.012	1.92E-03	9.95E-05	1.027	1.10E-03	-0.001	0.007	9.59E-04	0.000	0.000	1.08E-03	0.000	0.005	3.00E-04
6	2.32E-03	8.55E-05	1.023	1.88E-03	1.04E-04	1.024	1.90E-03	9.80E-05	1.034	1.23E-03	-0.004	0.005	1.04E-03	0.001	-0.005	1.14E-03	-0.002	0.006	4.89E-04
7	2.20E-03	8.61E-05	1.032	1.95E-03	1.05E-04	1.012	2.07E-03	9.40E-05	1.021	1.26E-03	-0.004	0.006	9.28E-04	0.003	-0.004	1.11E-03	-0.004	0.005	4.21E-04
8	2.33E-03	8.28E-05	1.026	1.94E-03	1.04E-04	1.018	2.05E-03	9.23E-05	1.031	1.12E-03	0.000	0.000	1.08E-03	0.000	0.000	1.07E-03	-0.008	0.008	2.52E-04
9	2.24E-03	8.76E-05	1.024	1.85E-03	1.06E-04	1.021	1.97E-03	9.71E-05	1.027	1.12E-03	-0.002	0.003	8.10E-04	0.001	-0.005	1.01E-03	-0.002	0.004	2.78E-04
10	2.23E-03	8.70E-05	1.027	1.84E-03	1.10E-04	1.016	2.11E-03	9.38E-05	1.021	1.12E-03	-0.003	0.006	9.72E-04	0.002	-0.004	1.09E-03	-0.001	0.003	3.62E-04
11	2.27E-03	8.57E-05	1.026	1.87E-03	1.07E-04	1.019	1.89E-03	1.01E-04	1.028	1.17E-03	-0.004	0.007	8.28E-04	0.002	-0.005	9.69E-04	0.000	0.001	2.74E-04
12	2.15E-03	8.71E-05	1.034	1.93E-03	1.02E-04	1.019	2.05E-03	9.41E-05	1.025	1.24E-03	-0.001	0.005	9.36E-04	0.000	0.000	1.11E-03	-0.004	0.006	4.25E-04
13	2.26E-03	8.65E-05	1.023	1.82E-03	1.07E-04	1.024	1.93E-03	9.78E-05	1.029	1.16E-03	-0.005	0.008	9.08E-04	0.002	-0.005	9.69E-04	-0.002	0.004	3.09E-04
14	2.28E-03	8.92E-05	1.021	1.99E-03	1.02E-04	1.013	2.00E-03	9.81E-05	1.024	1.11E-03	-0.003	0.003	9.36E-04	0.002	-0.005	1.05E-03	0.000	0.000	1.63E-04
15	2.15E-03	8.96E-05	1.028	1.99E-03	9.90E-05	1.016	1.92E-03	1.01E-04	1.026	1.16E-03	-0.006	0.007	9.21E-04	0.000	0.000	1.05E-03	-0.003	0.006	2.22E-04
16	2.19E-03	8.64E-05	1.032	1.88E-03	1.06E-04	1.017	1.96E-03	9.64E-05	1.031	1.14E-03	-0.002	0.006	9.85E-04	0.002	-0.005	1.09E-03	-0.002	0.008	3.49E-04

Table S3. Parameter estimates for 16 independent MiXeR runs for the "equilibrium" simulated scenario.
The first column show the index of the optimization run (1-16), with the run having the smallest deviation from the median overlap pattern (as described in the methods section) marked with asterisk. Other columns show univariate polygenicities ($\pi_1^u, \pi_2^u, \pi_3^u$), discoverabilities ($\sigma_1, \sigma_2, \sigma_3$) and residual variances ($\sigma_{01}, \sigma_{02}, \sigma_{03}$) for Trait 1, Trait 2 and Trait 3 respectively; pairwise (bivariate) genetic overlaps ($\pi_{12}^b, \pi_{13}^b, \pi_{23}^b$), correlations of effect sizes within each of the three pairwise overlaps ($\rho_{12}, \rho_{13}, \rho_{23}$) and correlations between residuals ($\rho_{012}, \rho_{013}, \rho_{023}$) for Trait 1 and Trait 2, Trait 1 and Trait 3 and Trait 2 and Trait 3 pairs respectively; the genetic overlap between all three phenotypes (π_{123}).

Index	π_1^u	σ_2	σ_{01}	π_2^u	σ_2	σ_{02}	π_3^u	σ_3	σ_{03}	π_{12}^b	ρ_{12}	ρ_{012}	π_{13}^b	ρ_{13}	ρ_{013}	π_{23}^b	ρ_{23}	ρ_{023}	π_{123}
1	1.99E-03	9.14E-05	1.035	1.95E-03	9.73E-05	1.027	2.02E-03	9.22E-05	1.030	1.01E-03	0.002	-0.003	1.26E-03	0.002	-0.003	9.45E-04	-0.010	0.009	5.29E-04
2	2.11E-03	8.61E-05	1.032	2.02E-03	9.24E-05	1.030	1.97E-03	9.66E-05	1.026	1.04E-03	0.002	-0.006	1.19E-03	0.002	-0.006	8.77E-04	-0.002	0.005	5.22E-04
3	2.04E-03	8.97E-05	1.034	2.12E-03	8.84E-05	1.027	2.07E-03	9.29E-05	1.026	1.15E-03	0.002	-0.007	1.17E-03	0.000	0.000	1.06E-03	-0.005	0.005	6.30E-04
4	2.10E-03	8.69E-05	1.035	2.06E-03	9.29E-05	1.027	1.97E-03	9.26E-05	1.034	1.01E-03	0.002	-0.007	1.30E-03	0.002	-0.004	9.15E-04	-0.003	0.004	8.37E-04
5	2.13E-03	8.52E-05	1.032	2.03E-03	8.94E-05	1.036	1.88E-03	9.98E-05	1.029	1.24E-03	0.002	-0.007	1.32E-03	0.001	-0.001	7.70E-04	-0.004	0.006	5.44E-04
6	2.09E-03	8.89E-05	1.030	1.98E-03	8.99E-05	1.038	2.12E-03	9.16E-05	1.022	1.05E-03	0.002	-0.004	1.31E-03	0.000	0.000	8.53E-04	0.000	0.000	3.72E-04
7	2.16E-03	8.35E-05	1.034	1.96E-03	9.13E-05	1.038	2.17E-03	8.65E-05	1.031	1.09E-03	0.001	-0.001	1.40E-03	0.001	-0.003	9.22E-04	0.000	0.000	8.60E-04
8	2.06E-03	8.57E-05	1.039	2.00E-03	8.72E-05	1.042	1.92E-03	9.77E-05	1.033	1.18E-03	0.001	-0.007	1.18E-03	0.001	-0.001	9.62E-04	-0.005	0.005	7.60E-04
9	1.98E-03	9.36E-05	1.030	2.12E-03	8.99E-05	1.025	2.11E-03	9.21E-05	1.024	1.18E-03	0.003	-0.008	1.37E-03	0.001	-0.002	1.03E-03	-0.005	0.005	7.87E-04
10	1.87E-03	9.55E-05	1.040	2.02E-03	9.03E-05	1.035	2.06E-03	9.15E-05	1.031	9.49E-04	0.002	-0.007	1.15E-03	0.000	-0.001	1.02E-03	-0.010	0.010	4.94E-04
11*	2.00E-03	8.86E-05	1.039	2.05E-03	9.12E-05	1.026	1.99E-03	9.46E-05	1.028	1.14E-03	0.002	-0.005	1.23E-03	0.000	0.000	8.84E-04	-0.003	0.006	5.56E-04
12	2.10E-03	8.70E-05	1.034	2.07E-03	9.00E-05	1.027	2.03E-03	9.31E-05	1.030	1.03E-03	0.003	-0.008	1.30E-03	0.005	-0.010	8.93E-04	-0.003	0.006	5.21E-04
13	2.03E-03	8.82E-05	1.037	2.07E-03	9.01E-05	1.030	2.07E-03	9.15E-05	1.031	1.14E-03	0.001	-0.006	1.37E-03	0.002	-0.006	9.13E-04	-0.002	0.004	7.33E-04
14	2.06E-03	8.56E-05	1.041	2.01E-03	9.00E-05	1.034	2.15E-03	9.10E-05	1.023	1.12E-03	0.006	-0.008	1.31E-03	0.002	-0.008	9.41E-04	-0.003	0.003	5.74E-04
15	2.04E-03	8.67E-05	1.038	2.05E-03	8.85E-05	1.033	2.12E-03	9.33E-05	1.020	1.16E-03	0.002	-0.007	1.24E-03	0.000	-0.004	9.75E-04	-0.010	0.010	7.01E-04
16	2.13E-03	8.48E-05	1.034	2.23E-03	8.62E-05	1.023	1.92E-03	9.88E-05	1.028	1.13E-03	0.005	-0.013	1.24E-03	0.000	0.000	9.26E-04	-0.004	0.003	5.97E-04

Table S4. Parameter estimates for 16 independent MiXeR runs for type 2 diabetes (T2D), high-density lipoprotein (HDL) and estimated glomerular filtration rate (eGFR). The first column show the index of the optimization run (1-16), with the run having the smallest deviation from the median overlap pattern (as described in the methods section) marked with asterisk. Other columns show univariate polygenicities ($\pi_1^u, \pi_2^u, \pi_3^u$), discoverabilities ($\sigma_1, \sigma_2, \sigma_3$) and residual variances ($\sigma_{01}, \sigma_{02}, \sigma_{03}$) for T2D, HDL and eGFR respectively; pairwise (bivariate) genetic overlaps ($\pi_{12}^b, \pi_{13}^b, \pi_{23}^b$), correlations of effect sizes within each of the three pairwise overlaps ($\rho_{12}, \rho_{13}, \rho_{23}$) and correlations between residuals ($\rho_{012}, \rho_{013}, \rho_{023}$) for T2D and HDL, T2D and eGFR and HDL and eGFR pairs respectively; the genetic overlap between all three phenotypes (π_{123}).

Index	π_1^u	σ_2	σ_{01}	π_2^u	σ_2	σ_{02}	π_3^u	σ_3	σ_{03}	π_{12}^b	ρ_{12}	ρ_{012}	π_{13}^b	ρ_{13}	ρ_{013}	π_{23}^b	ρ_{23}	ρ_{023}	π_{123}
1	5.18E-04	1.30E-04	1.066	3.62E-04	1.80E-04	1.276	4.16E-04	8.45E-05	1.058	2.75E-04	-0.411	-0.228	1.52E-04	0.217	0.000	1.51E-04	0.096	0.003	1.35E-04
2*	5.16E-04	1.30E-04	1.066	3.81E-04	1.69E-04	1.268	4.27E-04	8.31E-05	1.058	2.66E-04	-0.429	-0.227	1.70E-04	0.221	0.000	1.60E-04	0.146	0.003	1.24E-04
3	4.58E-04	1.48E-04	1.067	3.71E-04	1.75E-04	1.276	4.03E-04	8.50E-05	1.065	1.68E-04	-0.679	-0.219	1.64E-04	0.077	0.000	1.02E-04	0.218	0.000	1.02E-04
4	4.85E-04	1.39E-04	1.066	3.87E-04	1.68E-04	1.268	3.85E-04	9.01E-05	1.065	2.78E-04	-0.503	-0.222	1.52E-04	0.156	0.000	1.44E-04	0.128	0.001	1.31E-04
5	4.83E-04	1.39E-04	1.066	4.06E-04	1.57E-04	1.255	4.05E-04	8.74E-05	1.061	3.22E-04	-0.225	-0.219	1.39E-04	0.216	0.000	1.54E-04	0.154	0.000	1.05E-04
6	4.75E-04	1.37E-04	1.079	3.70E-04	1.74E-04	1.276	3.96E-04	8.81E-05	1.066	2.55E-04	-0.300	-0.210	2.09E-04	0.077	0.000	1.20E-04	0.150	0.000	1.20E-04
7	4.98E-04	1.34E-04	1.066	3.72E-04	1.73E-04	1.276	4.01E-04	8.36E-05	1.072	1.77E-04	-0.764	-0.222	1.59E-04	0.146	0.000	1.52E-04	0.144	0.000	5.57E-05
8	5.21E-04	1.31E-04	1.066	3.78E-04	1.65E-04	1.281	4.43E-04	7.93E-05	1.057	2.73E-04	-0.394	-0.223	1.79E-04	0.090	0.002	1.49E-04	0.220	0.000	1.27E-04
9	5.04E-04	1.34E-04	1.066	3.70E-04	1.75E-04	1.276	4.11E-04	8.39E-05	1.064	1.65E-04	-0.764	-0.217	1.65E-04	0.139	0.000	1.46E-04	0.078	0.000	7.53E-05
10	5.12E-04	1.31E-04	1.066	3.83E-04	1.66E-04	1.276	4.03E-04	8.75E-05	1.062	1.77E-04	-0.896	-0.222	1.53E-04	0.213	0.000	1.42E-04	0.219	0.000	7.32E-05
11	5.47E-04	1.30E-04	1.048	3.75E-04	1.70E-04	1.276	4.19E-04	8.44E-05	1.058	2.21E-04	-0.618	-0.222	1.28E-04	0.216	0.000	1.53E-04	0.155	0.001	1.28E-04
12	4.94E-04	1.30E-04	1.078	3.69E-04	1.70E-04	1.276	4.05E-04	8.71E-05	1.062	2.14E-04	-0.529	-0.209	1.49E-04	0.223	0.000	1.42E-04	0.156	0.000	3.80E-05
13	4.95E-04	1.37E-04	1.065	3.80E-04	1.67E-04	1.276	3.98E-04	8.58E-05	1.068	1.76E-04	-0.777	-0.222	1.55E-04	0.151	0.000	1.49E-04	0.074	0.001	1.00E-06
14	4.61E-04	1.44E-04	1.072	3.81E-04	1.67E-04	1.276	4.00E-04	8.51E-05	1.070	1.48E-04	-0.847	-0.223	1.41E-04	0.220	0.000	2.04E-04	0.151	0.000	1.01E-04
15	4.60E-04	1.48E-04	1.069	3.66E-04	1.79E-04	1.276	3.93E-04	8.83E-05	1.065	2.73E-04	-0.322	-0.219	1.53E-04	0.144	0.000	1.14E-04	0.150	0.000	8.10E-05
16	4.54E-04	1.52E-04	1.066	3.77E-04	1.70E-04	1.276	4.01E-04	8.84E-05	1.055	2.93E-04	-0.439	-0.218	1.53E-04	-0.006	0.004	1.60E-04	0.065	0.006	8.68E-05

Table S5. Parameter estimates for 16 independent MiXeR runs for ulcerative colitis (UC), psoriasis (PS), and multiple sclerosis (MS).
The first column show the index of the optimization run (1-16), with the run having the smallest deviation from the median overlap pattern (as described in the methods section) marked with asterisk. Other columns show univariate polygenicities ($\pi_1^u, \pi_2^u, \pi_3^u$), discoverabilities ($\sigma_1, \sigma_2, \sigma_3$) and residual variances ($\sigma_{01}, \sigma_{02}, \sigma_{03}$) for UC, PS and MS respectively; pairwise (bivariate) genetic overlaps ($\pi_{12}^b, \pi_{13}^b, \pi_{23}^b$), correlations of effect sizes within each of the three pairwise overlaps ($\rho_{12}, \rho_{13}, \rho_{23}$) and correlations between residuals ($\rho_{012}, \rho_{013}, \rho_{023}$) for UC and PS, UC and MS and PS and MS pairs respectively; the genetic overlap between all three phenotypes (π_{123}).

Index	π_1^u	σ_2	σ_{01}	π_2^u	σ_2	σ_{02}	π_3^u	σ_3	σ_{03}	π_{12}^b	ρ_{12}	ρ_{012}	π_{13}^b	ρ_{13}	ρ_{013}	π_{23}^b	ρ_{23}	ρ_{023}	π_{123}
1	1.22E-04	1.23E-03	1.121	1.06E-04	7.62E-04	1.095	2.02E-04	8.39E-04	1.043	6.32E-05	0.192	0.011	8.12E-05	0.185	0.068	8.15E-05	0.222	0.000	6.32E-05
2	1.37E-04	1.12E-03	1.118	1.04E-04	7.75E-04	1.093	1.98E-04	8.83E-04	1.043	6.70E-05	0.156	0.011	9.18E-05	0.217	0.066	7.96E-05	0.212	0.000	6.70E-05
3	1.51E-04	1.01E-03	1.117	1.07E-04	7.55E-04	1.094	1.82E-04	9.07E-04	1.049	6.80E-05	0.227	0.010	9.09E-05	0.212	0.064	8.33E-05	0.184	0.004	6.80E-05
4	1.17E-04	1.27E-03	1.122	1.40E-04	6.32E-04	1.087	1.87E-04	9.00E-04	1.047	6.59E-05	0.200	0.015	7.57E-05	0.224	0.064	9.31E-05	0.211	0.000	6.59E-05
5	1.15E-04	1.31E-03	1.121	8.61E-05	8.94E-04	1.095	1.90E-04	9.28E-04	1.043	5.68E-05	0.140	0.012	7.92E-05	0.203	0.064	6.82E-05	0.220	0.000	5.68E-05
6	1.33E-04	1.15E-03	1.117	8.46E-05	8.81E-04	1.096	1.96E-04	8.61E-04	1.047	5.76E-05	0.138	0.014	8.98E-05	0.207	0.065	6.92E-05	0.180	0.004	5.76E-05
7	1.26E-04	1.21E-03	1.116	1.45E-04	5.89E-04	1.089	2.06E-04	8.40E-04	1.046	7.57E-05	0.215	0.009	8.19E-05	0.238	0.063	9.90E-05	0.231	0.000	7.57E-05
8	1.06E-04	1.35E-03	1.126	1.15E-04	7.15E-04	1.089	1.87E-04	9.06E-04	1.045	6.37E-05	0.205	0.011	6.89E-05	0.209	0.066	8.40E-05	0.203	0.001	6.37E-05
9	1.14E-04	1.25E-03	1.124	1.06E-04	7.40E-04	1.094	2.17E-04	8.16E-04	1.039	6.87E-05	0.174	0.005	8.06E-05	0.205	0.065	8.43E-05	0.165	0.001	6.87E-05
10*	1.20E-04	1.23E-03	1.122	1.17E-04	6.88E-04	1.090	2.02E-04	8.71E-04	1.038	6.18E-05	0.199	0.010	7.81E-05	0.249	0.064	9.06E-05	0.169	0.001	6.18E-05
11	1.20E-04	1.23E-03	1.119	1.22E-04	6.54E-04	1.093	1.87E-04	8.68E-04	1.051	7.43E-05	0.000	0.014	8.31E-05	0.221	0.065	9.43E-05	0.161	0.002	7.43E-05
12	1.18E-04	1.28E-03	1.121	1.36E-04	6.30E-04	1.091	1.99E-04	8.53E-04	1.045	8.06E-05	0.197	0.001	7.73E-05	0.225	0.067	8.97E-05	0.209	0.000	7.73E-05
13	1.11E-04	1.33E-03	1.125	1.03E-04	7.87E-04	1.094	1.69E-04	1.03E-03	1.048	6.11E-05	0.152	0.015	7.06E-05	0.234	0.064	3.17E-05	0.439	0.000	3.17E-05
14	1.21E-04	1.22E-03	1.120	1.09E-04	6.98E-04	1.095	1.69E-04	1.01E-03	1.049	7.10E-05	0.188	0.015	6.36E-05	0.354	0.067	8.73E-05	0.179	0.001	6.15E-05
15	1.15E-04	1.28E-03	1.125	1.21E-04	6.71E-04	1.092	2.08E-04	8.60E-04	1.042	7.26E-05	0.230	0.008	8.56E-05	0.214	0.066	9.93E-05	0.175	0.003	7.26E-05
16	1.19E-04	1.23E-03	1.123	1.12E-04	7.14E-04	1.091	2.16E-04	8.01E-04	1.044	6.94E-05	0.152	0.012	9.23E-05	0.212	0.061	9.17E-05	0.211	0.001	6.94E-05

Table S6. Parameter estimates for 16 independent MiXeR runs for placental weight (PW), schizophrenia (SCZ) and height.
The first column show the index of the optimization run (1-16), with the run having the smallest deviation from the median overlap pattern (as described in the methods section) marked with asterisk. Other columns show univariate polygenicities ($\pi_1^u, \pi_2^u, \pi_3^u$), discoverabilities ($\sigma_1, \sigma_2, \sigma_3$) and residual variances ($\sigma_{01}, \sigma_{02}, \sigma_{03}$) for PW, SCZ and height respectively; pairwise (bivariate) genetic overlaps ($\pi_{12}^b, \pi_{13}^b, \pi_{23}^b$), correlations of effect sizes within each of the three pairwise overlaps ($\rho_{12}, \rho_{13}, \rho_{23}$) and correlations between residuals ($\rho_{012}, \rho_{013}, \rho_{023}$) for PW and SCZ, PW and height and SCZ and height pairs respectively; the genetic overlap between all three phenotypes (π_{123}).

Index	π_1^u	σ_2	σ_{01}	π_2^u	σ_2	σ_{02}	π_3^u	σ_3	σ_{03}	π_{12}^b	ρ_{12}	ρ_{012}	π_{13}^b	ρ_{13}	ρ_{013}	π_{23}^b	ρ_{23}	ρ_{023}	π_{123}
1*	3.91E-04	1.74E-04	1.038	2.88E-03	6.06E-05	1.155	1.28E-03	1.81E-04	2.096	7.11E-05	-0.233	0.000	2.83E-04	0.557	0.037	2.80E-04	-0.157	-0.023	7.11E-05
2	3.93E-04	1.76E-04	1.035	2.95E-03	5.94E-05	1.158	1.26E-03	1.82E-04	2.126	1.08E-04	0.002	-0.005	2.95E-04	0.531	0.038	2.42E-04	-0.311	-0.011	1.08E-04
3	3.89E-04	1.72E-04	1.042	2.81E-03	6.21E-05	1.157	1.27E-03	1.83E-04	2.108	4.81E-05	-0.228	-0.008	3.10E-04	0.510	0.035	3.04E-04	-0.145	-0.017	3.72E-05
4	4.19E-04	1.62E-04	1.040	2.93E-03	6.13E-05	1.145	1.25E-03	1.86E-04	2.122	1.51E-05	1.000	-0.006	3.09E-04	0.507	0.039	3.04E-04	-0.151	-0.016	1.00E-06
5	3.98E-04	1.71E-04	1.037	2.85E-03	6.19E-05	1.153	1.26E-03	1.86E-04	2.106	8.03E-05	0.002	-0.008	2.96E-04	0.540	0.037	2.98E-04	-0.166	-0.012	8.03E-05
6	3.68E-04	1.77E-04	1.045	2.84E-03	6.15E-05	1.158	1.24E-03	1.86E-04	2.125	7.57E-05	0.004	-0.004	2.70E-04	0.556	0.038	3.04E-04	-0.135	-0.020	7.57E-05
7	4.15E-04	1.63E-04	1.041	2.81E-03	6.26E-05	1.154	1.26E-03	1.84E-04	2.105	8.05E-06	0.889	-0.001	3.01E-04	0.549	0.035	2.57E-04	-0.296	-0.002	1.00E-06
8	4.13E-04	1.66E-04	1.039	2.87E-03	6.16E-05	1.153	1.27E-03	1.82E-04	2.099	7.34E-05	-0.229	0.000	3.03E-04	0.516	0.038	2.79E-04	-0.289	-0.002	7.34E-05
9	3.56E-04	1.80E-04	1.044	2.88E-03	6.09E-05	1.154	1.23E-03	1.89E-04	2.137	9.62E-05	0.003	-0.006	2.70E-04	0.545	0.038	3.13E-04	-0.224	0.000	9.62E-05
10	4.16E-04	1.61E-04	1.039	2.95E-03	5.97E-05	1.151	1.26E-03	1.84E-04	2.111	5.31E-05	-0.267	-0.004	3.06E-04	0.535	0.036	2.44E-04	-0.349	-0.004	5.31E-05
11	3.69E-04	1.85E-04	1.038	2.98E-03	5.91E-05	1.154	1.25E-03	1.86E-04	2.126	2.25E-05	0.775	-0.008	2.77E-04	0.508	0.038	3.17E-04	-0.123	-0.016	1.21E-05
12	3.63E-04	1.84E-04	1.041	2.78E-03	6.25E-05	1.158	1.23E-03	1.88E-04	2.125	9.21E-05	0.003	-0.006	2.74E-04	0.538	0.038	2.89E-04	-0.144	-0.020	9.21E-05
13	3.94E-04	1.69E-04	1.041	2.78E-03	6.27E-05	1.159	1.26E-03	1.83E-04	2.117	1.02E-04	0.003	-0.008	2.99E-04	0.527	0.033	2.10E-04	-0.357	-0.013	1.02E-04
14	3.30E-04	1.93E-04	1.045	3.10E-03	5.74E-05	1.148	1.24E-03	1.87E-04	2.125	1.57E-05	0.969	-0.006	2.65E-04	0.542	0.037	3.51E-04	-0.145	-0.009	1.00E-06
15	3.89E-04	1.73E-04	1.040	2.86E-03	6.08E-05	1.156	1.26E-03	1.84E-04	2.090	9.51E-05	0.002	-0.006	2.91E-04	0.534	0.036	1.08E-04	-0.652	-0.016	9.51E-05
16	3.68E-04	1.78E-04	1.043	2.90E-03	5.98E-05	1.159	1.26E-03	1.82E-04	2.117	1.41E-05	0.882	-0.004	2.78E-04	0.507	0.039	2.82E-04	-0.167	-0.014	1.70E-06
